# Case report: cardiac herniation following robotic-assisted thymectomy

**DOI:** 10.1186/s13019-020-01093-3

**Published:** 2020-03-30

**Authors:** John Espey, Stephen Acosta, Lavinia Kolarczyk, Jason Long

**Affiliations:** 1 Department of Cardiothoracic Surgery, University of North Carolina-Chapel Hill, 101 Manning Drive, Chapel Hill, NC 27705 USA; 2 Department of Anesthesiology, University of North Carolina-Chapel Hill, 101 Manning Drive, Chapel Hill, NC 27705 USA

**Keywords:** Thymectomy, Cardiac herniation, Robotic-assisted, Minimally invasive, Thymoma, Myasthenia gravis

## Abstract

**Background:**

The first reported case of cardiac herniation was in 1948 and occurred following pericardiectomy during a lung cancer resection. Although rare, this potentially fatal surgical complication may occur following any operation in which a pericardial incision or resection is performed. The majority of literature on cardiac herniation involves case reports after intrapericardial pneumonectomy. Currently, there are no reports of cardiac herniation after thymectomy with pericardial resection.

**Case presentation:**

A 44-year-old Asian female with symptomatic myasthenia gravis was referred for thymectomy. Originally thought to have Bell’s Palsy, her symptoms began with right eyelid drooping and facial weakness. Over time, she developed difficulty holding her head up, upper extremity weakness, difficulty chewing and dysarthria. These symptoms worsened with activity. She was found to have positive acetylcholine receptor binding antibody on her myasthenia gravis panel. A preoperative CT scan demonstrated a 3.5 cm × 2 cm anterior mediastinal mass along the right heart border and phrenic nerve. A complete thymectomy, via right-sided robotic-assisted approach was performed en bloc with a portion of the right phrenic nerve and a 4 cm × 4 cm portion of pericardium overlying the right atrium and superior right ventricle. Upon undocking of the robot and closure of the port sites, the patient became acutely hypotensive (lowest recorded blood pressure 43/31 mmHg). The camera was reinserted and demonstrated partial cardiac herniation through the anterior pericardial defect toward the right chest. An emergent midline sternotomy was performed and the heart was manually reduced. The patient’s hemodynamics stabilized. A vented Gore-Tex 6 cm × 6 cm patch was sewn along the pericardial edges with interrupted 4–0 prolene to close the pericardial defect.

**Conclusion:**

This potentially fatal complication, although rare, should always be considered whenever there is hemodynamic instability entry or resection of the pericardium during surgery. We now routinely sew in a pericardial patch using our robotic surgical system for any defect over 3 cm × 3 cm that extends from the mid- to inferior portions of the heart.

## Background

Cardiac herniation is a rare but potentially fatal complication encountered in general thoracic surgery. The first reported case of cardiac herniation was in 1948 by Bettman et al. [1]. Although usually reported with blunt chest trauma or pneumonectomy, cardiac herniation is possible with any procedure involving pericardial incision or resection. We present a case of acute cardiac herniation after a right-sided robotic-assisted thymectomy in which a portion of the pericardium was resected.

## Case presentation

**Fig. 1:**
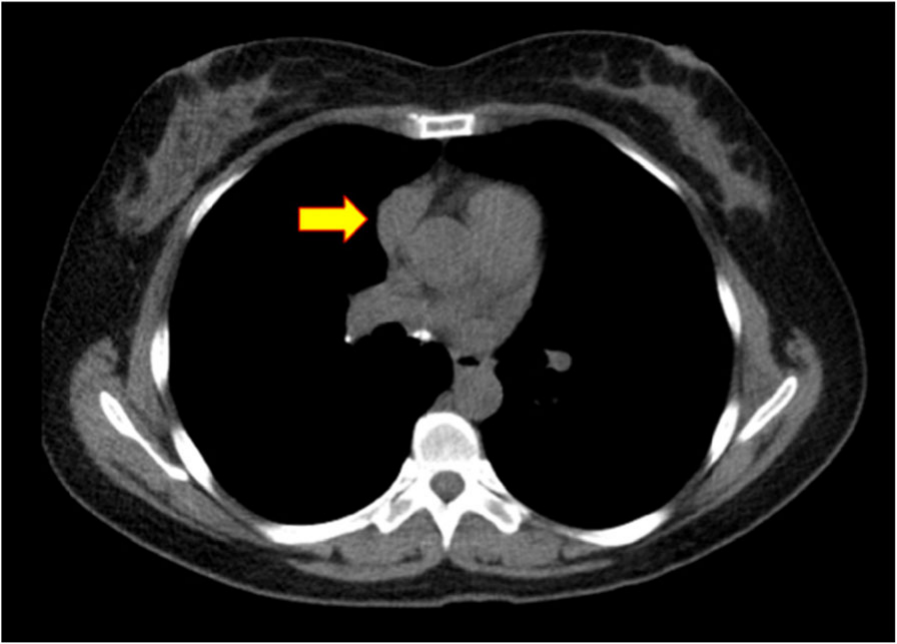
Preoperative CT of 3.5 cm × 2 cm anterior mediastinal mass along the right heart border and phrenic nerve

44-year-old Asian female with history of symptomatic myasthenia gravis was referred for a thymectomy. She was originally believed to have Bell’s palsy due to drooping of her right eyelid and facial weakness. Her symptoms worsened as she developed difficulty holding her head up, weakness in her arms, difficultly chewing and dysarthria. Symptoms worsened with activity. Her myasthenia gravis panel was positive for acetylcholine receptor binding antibody 7.81 nmol/L (normal range < 0.02). Her preoperative CT scan demonstrated a 3.5 cm × 2 cm anterior mediastinal mass along the right heart border and phrenic nerve, suspicious for Masaoka Stage III thymoma (Fig. 1). No preoperative biopsy was performed. Her only medication was mycophenolate mofetil 500 mg BID. She had never been hospitalized for her myasthenia nor had a history of a crisis. At the discretion of her neurologist, she did not require IVIG treatment in advance of her operation. The patient was offered a thymectomy via right-sided robotic-assisted approach based on the location of the tumor on imaging [2].

**Fig. 2:**
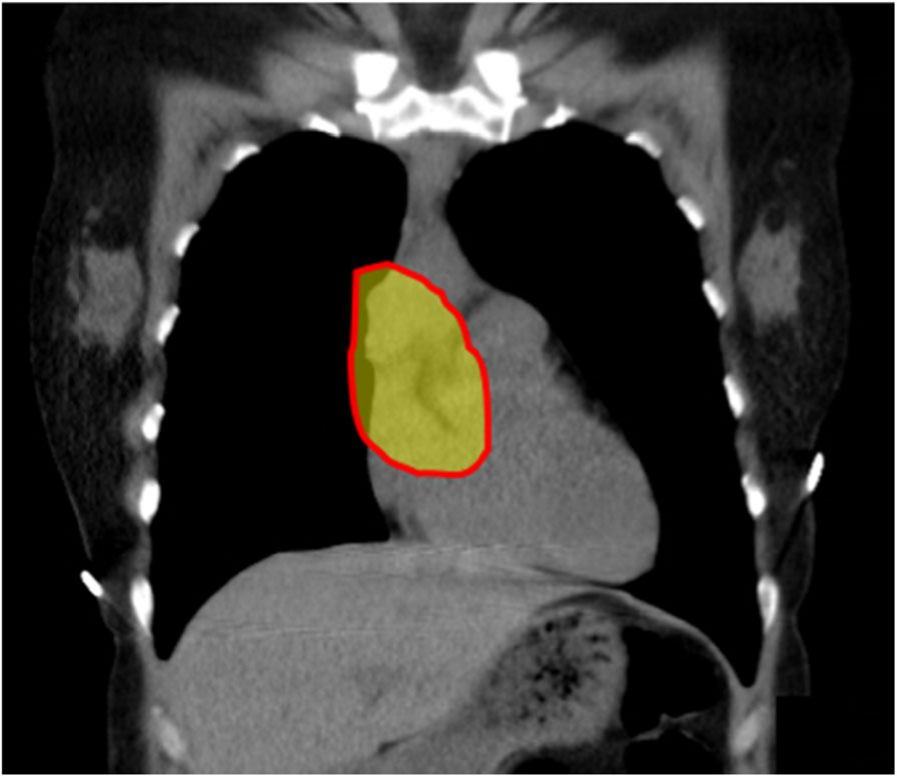
CT scan with highlighted region demonstrating location 4x4cm resection involving pericardium and portion of phrenic nerve

**Fig. 3:**
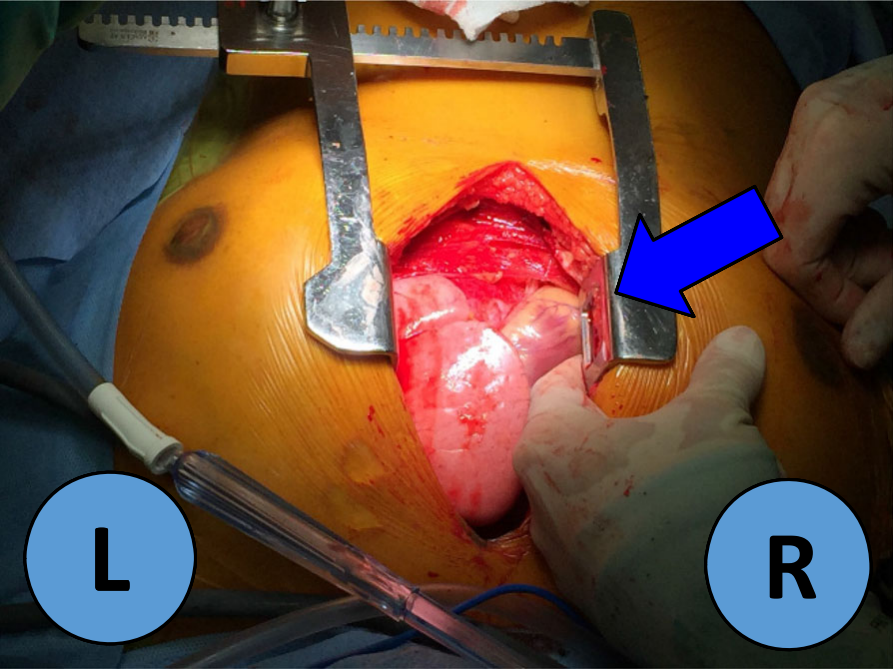
Intraoperative cardiac herniation from this operation. L = left, R = right, Blue Arrow: cardiac herniation

Following induction of general anesthesia, the patient was intubated with a left-sided double lumen endotracheal tube. She was positioned supine with a bump placed under the right chest and the right arm allowed to hang below the chest with appropriate padding. Right lung isolation was achieved. Three 8 mm robotic ports were placed in the right 2nd, 4th, and 6th intercostal spaces along the mid-axillary line. The daVinci Xi robot was docked and CO2 insufflation with a pressure of 12 mmHg was initiated permitting an adequate view of the right hemithorax as well as the anterior mediastinum. Upon initial inspection, a 3 cm × 3 cm mass was identified centered over the right atrial-SVC junction with invasion of the right phrenic nerve and pericardium. A complete thymectomy was performed en bloc with a portion of the right phrenic nerve and a 4 cm × 4 cm portion of pericardium overlying the right atrium and superior right ventricle (Fig. 2). The specimen was sent for frozen pathologic analysis and returned suspicious for thymoma. A 28Fr chest drain was placed across the mediastinum via her inferior-most port site. Hemostasis was achieved and the instruments and ports were removed. Upon undocking of the robot and closure of the port sites, the patient became acutely hypotensive (lowest recorded blood pressure 43/31 mmHg). The camera was reinserted and demonstrated partial cardiac herniation through the anterior pericardial defect toward the right chest. An emergent midline sternotomy was performed and a sternal retractor was placed (Fig. 3). The heart was manually reduced and the patient’s hemodynamics stabilized. A vented Gore-Tex 6 cm × 6 cm patch was sewn along the pericardial edges with interrupted 4–0 prolene to close the pericardial defect. The patient was transferred to the ICU postoperatively. The patient was extubated on postoperative day one and discharged home on postoperative day seven. The final pathology was a Masaoka Type 3 invasive type B3 thymoma.

## Discussion and conclusions

To the best of our knowledge, there are no reports on intra- or postoperative cardiac herniation following minimally invasive thymectomy with pericardial resection. The incidence of cardiac herniation is rare and most commonly associated with pneumonectomy. The laterality of the herniation depends on the side in which the pericardium was entered and/or resected [1]. The majority of literature consists of case reports after intrapericardial pneumonectomy where the defect in the pericardium is deliberately left open [3]. Herniation of the heart can occur with equal frequency after a right-sided or left sided pneumonectomy [4]. It has occurred in patients with both small and large pericardial defects as well as in patients who have undergone excision of the pericardium to prevent this complication [5]. In addition to iatrogenic causes, cardiac herniation may also be associated with congenital pericardial defects or after traumatic chest wall trauma.

The differential diagnosis of acute intraoperative hypotension during minimally invasive thoracic surgery includes CO2 insufflation impeding preload to the heart, tension pneumothorax, hemorrhage, myocardial infarction, pulmonary embolism, anaphylaxis, etc. Although cardiac herniation is a rare event, it must be considered with any operation involving a pericardial incision or resection [6]. Although the incidence is similar on both sides of the heart, the mechanism of hemodynamic failure differs [7]. A right sided herniation can present in a variety of ways: asymptomatic, severe hypotension, reflex tachycardia or superior vena cava syndrome (dependent on the amount of right ventricular compression). Left sided herniation is more likely to present with arrhythmia and myocardial ischemia due to compression of the left ventricular wall [8, 9]. Overall, these symptoms are most likely to occur within 24 h after surgery with 75% occurring at the end of the operation with patient repositioning [10]. Factors that increase the likelihood of cardiac herniation include size of pericardial defect, increased intrathoracic pressure due to excessive ventilation of contralateral lung, coughing on extubation or excessive suction from the ipsilateral chest tube. Currently, there are no specific size criteria established necessitating closure of a pericardial defect.

Some authors advise to close all pericardial defects, regardless of size. In one specific case a 4 cm × 4 cm “low likelihood” iatrogenic pericardial defect near the SVC and right main bronchus expanded to 7x5cm resulting in herniation [1]. However, even with repair, cardiac herniation should still be on differential as there is possibility of suture dehiscence with herniation following repair [11–13].

To date, no studies have compared the likelihood of cardiac herniation using minimally invasive vs open surgery techniques. However, it can be postulated that the use of intraoperative CO2 insufflation may have prevented herniation until the conclusion of the case when insufflation was discontinued and negative pressure was restored in the chest following port site closure. This, in conjunction with repositioning of the patient from a semi-lateral to a supine position, likely increased the likelihood herniation. Although cardiac herniation is a rare occurrence, we now routinely sew in a pericardial patch using our robotic surgical system for any defect over 3 cm × 3 cm that extends from the mid- to inferior portions of the heart. Using a 0.1 mm thickness PTFE membrane or bovine pericardium, the pericardial defect is repaired with interrupted 2–0 non-absorbable sutures intracorporeally using the robotic needle drivers. Small holes are cut in the pericardial patch to drain fluid. The robotic-assisted approach provides the necessary dexterity to not only perform an adequate thymectomy but a patch repair of a pericardial defect with interrupted suture. During the pericardial reconstruction phase, all sutures can be visualized from various angles, leading to a higher likelihood that the repair is adequate [5].

The authors acknowledge that this event was predictable given the size and location of the defect and should have never occurred. We present this case for its educational value. Based on the events of the described case, we recommend repair of all pericardial defects greater than 3 cm × 3 cm that expose more than one half of either ventricle and/or exhibit obvious prolapse of the cardiac tissue. It is important to realize that not every defect needs to be repaired as smaller defects above the A-V groove are at extremely low risk of herniation. However, in these particular cases, it is imperative that surgeons inspect the heart for herniation during CO2 exsufflation, when intra-thoracic pressure changes from positive to negative, and with repositioning the patient back to supine prior to removing the camera. In conclusion, cardiac herniation requires more research and investigation to establish guidelines for when a pericardial defect necessitates closure.

## Data Availability

Not Applicable.
